# “Life Starts for Me Again.” The Social Impact of Psychology on Programs for Homeless People: Solidarity Networks for the Effectiveness of Interventions

**DOI:** 10.3389/fpsyg.2019.03069

**Published:** 2020-02-04

**Authors:** Virginia Matulič-Domadzič, Ariadna Munté-Pascual, Irene De Vicente-Zueras, Susana León-Jiménez

**Affiliations:** ^1^UFR Escola de Treball Social, University of Barcelona, Barcelona, Spain; ^2^Department of Sociology, University of Barcelona, Barcelona, Spain

**Keywords:** homelessness, substance abuse, emotional support, intervention programs, psychologist, solidarity, potential social impact

## Abstract

The role of psychology in the improvement of people’s lives is consistent, according to the scientific literature review. More and more studies within psychology, and other social sciences, are pointing out the importance of the quality of social interactions on physical and mental health and upon perceived wellbeing. When talking about homelessness, psychology has served these individuals mostly by informing intervention programs related to preventing and responding to substance abuse, healthcare, sexual risks, or mental illnesses, and these are key problems that psychology has also studied and found to be connected to homelessness. Such strategies, which were traditionally mostly centered on tackling the weaknesses that homeless people face, are now increasingly considering the role of the social support services. The aim of this study was, on the one hand, to identify evidence on the importance of solidarity as a key factor in the process of overcoming homelessness, and associated substance abuse situations, and the barriers and conditions to achieve it. On another hand, the aim was also to approach the impact of that solidarity on their general wellbeing from the perspective of homeless subjects themselves. To achieve this aim, 20 life stories of people aged 35 to 70 years old were conducted in the metropolitan area of Barcelona. The people interviewed had been homeless in a recent period of their lives and were currently dependent on different services. The communicative methodology allowed deepening into the life stories of these individuals via egalitarian dialogue between the researchers and the participants. The results of the qualitative analysis showed that a strong solidarity network was crucial in the process of overcoming the participants’ homelessness situation and to tackle related circumstances (alcoholism and drug abuse among others), and this has had an impact in their general wellbeing and in the development of more solidarity attitudes. These findings are discussed in light of psychological knowledge and other social sciences literature on the importance of quality social environments to support positive developmental trajectories and considering the potential social impact of the intervention programs that take into account the factor of solidarity during their implementation process.

## Introduction

The role that psychology has in the improvement of people’s lives is undeniable and pervasive in the literature on psychological research. Contributions from psychology are crucial to the understanding and improvement of the living conditions of people living in complex situations that profoundly affect their wellbeing and quality of life, such as people in situations of homelessness. Research within psychology in the last decades has contributed to further the understanding of the phenomenon of homelessness, and it has also clarified associated causes, consequences, and related risks as well as preventive and mitigating factors. Being male, having low educational levels, being unemployed, and being separated from family at an early age have been identified as risk factors that can lead to homelessness, although, within homeless people, children, youth, and women are considered the most vulnerable groups (Browne, 1993; Philippot et al., 2007). Homelessness is associated with poor physical and mental health, and there is a high prevalence of substance abuse and dependence; in many cases, this was already a problem before the onset of homelessness (Philippot et al., 2007).

If one considers social impact to infer social improvements attained from the transference of the research results (Pulido et al., 2018), the social impact of psychology has served homeless people mostly by informing intervention programs related to preventing and responding to substance abuse (Davidson et al., 2013), healthcare (Salem and Ma-Pham, 2015; Fajardo-Bullón et al., 2019), sexual risks (Pedersen et al., 2018), or mental illnesses (Noël et al., 2016; Fond et al., 2019) – key problems that also psychology has studied and found to be connected to homelessness. Such strategies have often been centered on tackling the challenges that homeless people face, such as those mentioned above. However, homelessness is the result of the interaction of several factors, including socioeconomic factors and conditions of personal vulnerability (Shinn and Weitzman, 1990; Philippot et al., 2007).

Classical contributions of psychology have emphasized the multidimensionality of human wellbeing; they have helped further the understanding of the phenomenon of homelessness in which personal, interpersonal, and social dimensions interact as well as aided the analysis of the components that may contribute to overcoming it. This is the case of the humanist psychology, which stands out for its holistic approach to human existence. The contributions of Maslow (1943) and his pyramid of the hierarchy of needs to describe human motivations has particularly aided the understanding of the diversity of human needs, going from physiological needs to follow with safety, love, belonging, self-esteem, and finally self-actualization. Although Maslow’s original theory stated that higher levels in the hierarchy were pursued when a certain degree of the lower level needs were achieved, subsequent developments of the theory (Maslow, 1970), other authors’ contributions to this approach (Wahba and Bridwell, 1976), and research into the phenomenon of homelessness based on the theory (Henwood et al., 2015) take a less linear understanding and sustain the idea that also the frustration–and not only the fulfillment–of lower-level needs can lead to the pursuit of self-actualization, showing that there can be simultaneously different types of needs that are perceived and pursued, and these different needs are present at the same time, not only when the basic needs are covered.

Psychological theory also argues that the pursuit of higher-order needs can be hampered by sustained negative experiences of frustration, which could explain the maintenance of homelessness (Philippot et al., 2007). In this regard, the ideas of learned helplessness and self-efficacy (Bandura, 1997) are useful to explain this reality. Learned helplessness refers to the behavior of a person, after repeated negative events or situations not under their control, that results in abandoning efforts to escape the undesired situation. It is related to low self-efficacy, where self-efficacy is the personal judgment of “how well one can execute courses of action required to deal with prospective situations” (Bandura, 1982). Higher levels of self-efficacy are related to higher performance achievements and account for coping behavior, self-regulation of refractory behavior, resignation and despondency to failure experiences, and achievement strivings. Focusing on homelessness, Bandura (Epel et al., 1999) found that individuals with higher self-efficacy were more active in searching for housing and employment and had shorter stays in shelters, whereas individuals with lower self-efficacy tended to stay longer at the shelter.

This is related to the connection between homelessness and the experience of trauma. According to Goodman et al. (1991), for many homeless people, and especially for women, homelessness appears as a consequence of a situation entailing psychological trauma, such as physical or sexual abuse; furthermore, the presence of a high number of stressful events in their lives is a frequent occurrence in homeless people (Philippot et al., 2007). But homelessness itself is also itself a cause for the experience of psychological trauma because of the loss of one’s home and the conditions of shelter life (Goodman et al., 1991). The multiple trauma events experienced by homeless people would lead to the development of learned helplessness and low self-efficacy, preventing the overcoming of their situation. According to the authors, the prevention and reduction of the consequences of trauma can be achieved with a supportive and empowering environment, highlighting the importance of the social context and social relationships in fighting the negative psychological consequences of homelessness.

Resilience, as the ability to successfully adapt despite challenging or threatening circumstances, has also been studied within the context of homeless people (Masten et al., 1990; Paul et al., 2018). In this regard, research and interventions have focused on improving individuals’ resilience. Some interventions improved resilience and coping, focusing on raising awareness of personal character strengths and attitudes (Paul et al., 2018; Cooley et al., 2019). But far from being a purely individual way to react in difficult times, resilience is also related to social contexts and relationships.

According to Durbin et al. (2019), social support is related to increased resilience and reduced stress, thus minimizing the harmful consequences of stressful life events that homeless individuals face. The authors suggest the importance of interventions to help homeless people build support networks while measures are taken to improve their housing conditions. Paul et al. (2018) identified as coping strategies some that involved social relationships: seeking support from family, friends, and professionals; socializing with peers; engaging in meaningful activities; distancing from overwhelming challenges; and finding an anchor. Similarly, Miller and Bowen (2019) found the creation of support systems to be one of the attitudinal and behavior dimensions of resilience for homeless emerging adults. Other dimensions were perceptions of homelessness as a surmountable obstacle, externalization of homelessness, and the maintenance of personal health. Knight (2017) reported the use of group work to promote resilience among especially vulnerable groups of homeless people, such as homeless mothers, which facilitated sharing challenges, receiving support, and revealing participants’ strengths, which encouraged them to persevere to improve the situation they and their children were facing. For homeless adults with mental illness and substance use disorders, social support (either formal or informal) has been identified to be important for finding and maintaining housing (Gabrielian et al., 2018), and this has consequently highlighted the importance of developing practices that improve the social resources of homeless people. Social networks have also been related to the higher or lower risk for alcohol and other drugs consumption and have been the object of interventions that improve readiness to change the use of alcohol and other drugs and abstinence self-efficacy (Kennedy et al., 2017). Finally, social support has been promoted through physical activity; fitness interventions in the framework of supporting housing facilities (Sofija et al., 2018) and running groups for homeless women (Dawes et al., 2019) showed benefits both for physical and mental wellbeing and for social inclusion.

Through this body of research, it is shown how scientific literature is increasingly considering social support as a powerful element for overcoming the homelessness issue and how community-based interventions focused on social support can improve the personal resources for homeless people to cope and overcome their situation. Within the many forms that social support can take, *solidarity*, understood as the conjunction of every kind of prosocial behavior, such as being considerate, trustworthiness, cooperation, altruism, fairness, and not only to helping others but also a kind of sacrifice, has been widely studied and theorized from the point of view of psychology and other social sciences (Lindenberg, 2006). Research into community-based psychology has emphasized solidarity for its transformative potential to promote social change within vulnerable groups (Nelson and Evans, 2014). Less is known about the role that solidarity, as a specific form of social support, can have with regards to the homelessness issue. The purpose of this study was threefold: (a) to identify evidence regarding solidarity networks and attitudes as a key factor in the process of overcoming homelessness and related substance abuse situations; (b) to detect which barriers could hinder that solidarity and community support; and (c) to examine the impact of that solidarity on participants’ wellbeing from the perspective of homeless subjects themselves.

## Materials and Methods

### Design

The methodological design has been rooted in the Communicative Methodology, which is internationally recognized as a suitable method when working with groups of people in a vulnerable situation (Gómez et al., 2011; Puigvert et al., 2012; Gómez, 2019) and known for the political and social impact of the research that implements it (European Commission, 2010; Gómez and Jiménez, 2018). Communicative Methodology is based on egalitarian dialogue among researchers and the research end-users in all the stages of the research process, thus making it possible to construct knowledge from intersubjectivity and reflection among the different participants (Flecha, 2000; Lopez de Aguileta, 2019). This way, researchers’ biases are eliminated, and the benefits for the end-users increase, overcoming end-users’ concerns with other methodologies in which the researchers’ interests are prioritized over the social actors’ ones (Touraine et al., 2004; Flecha and Soler, 2014).

Reaching an effective implementation of the Communicative Methodology requires permeating the whole process with the aforementioned *egalitarian dialogue*, by which all contributions are considered according to validity claims (Habermas, 1989) instead of the power position of the participants (Flecha and Soler, 2014). Communicative Methodology incorporates within its analytical procedure two inherent components: the *exclusionary dimension*, entailing the identification of barriers, and the *transformative dimension*, which analyses the facilitators for reaching social transformation, i.e., the social impact (Gómez, 2017; García-Carrión et al., 2018; Gómez et al., 2019). Two main features of this methodology have led to its selection as the most appropriate method for the current study: (a) the way in which it finds the boundaries and enablers for a change that is consistent with the search for social impact and (b) an egalitarian and intersubjective dialogue among researchers and the participants that constitutes the sample of study. We needed a methodology that ensured the maximum openness of those men and women in a vulnerable situation, who had been victims of many adverse circumstances, in front of researchers given the distance of the social, academic, or economic realities of both parts, always with the stress being on the discovery of conditions for the social transformation of their realities through a common interpretation.

### Participants and Ethics

The research counted on the participation of 20 adult people, 14 women and 6 men, from 35 to 70 years old (with a high representation of people between the ages of 40 and 50), who had recently experienced–or where in process of overcoming–homelessness, and most of them were recovering from alcoholism and/or drug abuse. Regarding time experiencing homelessness, we found two kinds of situations: half of the people had lived on the street and another half of people had lived in temporary shelters and/or health services. The range of time for roofless participants covers from 3 weeks until 16 years with a higher representativity among 3 and 16 years. Participants who only lived in temporary accommodations covered a range from 1 to 4 years with the highest representativity between 1 and 2 years.

These people were recruited from the council’s social services and eight well-known public and private entities or non-profit organizations in Barcelona (Spain) located in different places of the metropolitan area (see Table 1). The recruitment of participants took part through snowball sampling, due to the previous relationships of researchers with the entities and particular professionals. The life stories were always conducted by a researcher in the entity or the accommodation of the participants, according to their convenience, and the goal was always to seek out natural interactions. Only the researcher and the interviewee were in the room during the interviews. They were audio recorded and subsequently transcribed.

**TABLE 1:** Organizations participants were affiliated to. Information collected from the official websites of the organizations.

Llar de Pau	A catholic social work program focused on women without resources in a situation of social exclusion. It is aimed at hosting and aiding in personal promotion toward the autonomy and the achievement of a personal life project.
Arrels	An organization that guides and assists the homeless people in Barcelona, providing coverage for health care, social or basic needs, and guaranteeing housing to the most vulnerable cases. They also work on the civil society and administrations awareness.
ProHabitatge	An independent non-profit association that works from a Human Rights angle with the aim of eliminating and preventing homelessness and residential exclusion in Catalonia. It runs housing and residential programs.
Cáritas	A charity organization founded by the Spanish Episcopal Conference with the aim of carrying out charitable and social actions through the promotion of an integral development, especially for the people most excluded in need.
Assís	A Reception Center with the objective of improving the wellbeing and quality of life of homeless people, especially women, through the implementation of projects and intervention programs focused on training, labor, health, basic needs and decent housing, and collaborating with other entities to search for sensibilization and an awareness of society.
Santa Lluïsa de Marillac	A social integration program from a Catholic charity aimed at people in a situation of social exclusion without resources that carries out attention actions through different project interventions: a welcome and counseling service; a day-care center; a center for limited housing for recovering homeless men; and housing for social inclusion.
Can Planas	Reception center in Barcelona for homeless adult people, which has the objective of covering basic needs through personalized help for the social insertion. It is focused on the development of skills and motivations of the people who are expected to lead a self-sufficient life once intervention in the center finishes. They also look for permanent resources for those people who need supervision or an adapted center indefinitely.
Sant Joan de Déu Solidarity	Non-profit religious entity aimed at supporting the fragile population of society (homeless people, mental illnesses, in dependency situation, childhood and youth, and disabilities) from three cornerstones: international cooperation, social work, and volunteering. It counts more than 400 social and sanitary centers in 55 countries.

The participants in the research were informed that their involvement was voluntary and anonymous and that all the data would be managed confidentially for solely research purposes. The ethical requirements were managed following the Ethics Review Procedure established by the European Commission (2013) for EU research, the Data Protection Directive 95/46/EC, and the Charter of Fundamental Rights of the European Union (2000/C 364/01). This study was entirely endorsed by the Ethics Board of the Community of Researchers on Excellence for All (CREA)^1^. To safeguard anonymity, original names of the participants has been substituted by pseudonyms when presenting the results of the study.

### Data Collection

Data were gathered through communicative life stories (Gómez et al., 2006; Gómez and Sordé, 2012) in order to delve deeper into the participants’ life trajectories from childhood until the present day. This was in order to have a complete understanding of every situation. For that purpose, a flexible interview guideline was used in order to ease the narrative and a comprehensive understanding, distinguishing the three stages of their life cycle (childhood, adolescence, and present day) while covering several topics transversally: familiar/love/social relationships, relations with professionals and friendships; economic situation; training and employment; housing; substance abuse; gender violence; what drove them to the organization; coexistence; effects of homelessness; wellbeing and health; coping mechanisms; and future prospects.

Each life story lasted around 2 h; some of them were conducted in a sole session, while others needed two sessions. These communicative life stories, where the subjects and the researchers, through an egalitarian dialogue, shared the life experiences of the end-users and the scientific knowledge of the researchers (Flecha and Soler, 2014), have helped to reveal interpretations and identify conditions and boundaries for the social transformation of this group of homeless men and women who participate in different organizations and programs with the purpose of normalizing their situation.

### Data Analysis

With the aim of the effective detection of the exclusionary and transformative dimensions in this study, i.e., the barriers and facilitators that homeless women and men find in their processes to transform their vulnerable circumstances through solidarity networks and a powerful social environment of support, the Communicative Methodology has been also implemented at the analysis stage. In order to follow the procedure of this methodology, with the information gathered through all the life stories, they have detected (a) an exclusionary dimension, according to all of the boundaries identified for the purpose of overcoming the cited vulnerable conditions of these homeless people, and (b) a transformative dimension, including the conditions that participants consider that have favored their process of recovery and the impact of solidarity on their general wellbeing.

Through the aforementioned intersubjective dialogue, the researcher and interviewees talked about the above topics during the communicative life story, pursuing the collection of the data needed for the analysis of both dimensions. The researcher and participants were at the same level, but the scientific background of the researcher allowed them to focus the conversation on the detection of barriers and facilitators. Once the information gathered, the analysis went on identifying both dimensions, exclusionary and transformative, for all categories. The categories coincided with topics proposed for the life stories, focusing especially on the coping mechanisms for overcoming homelessness and substance abuse and their impact in their general wellbeing.

## Results

The analysis carried out has shed light on the initial purposes of the research: finding the barriers that participants identify that impede achieving a stable situation regarding housing or detoxification; assessing the solidarity networks and attitudes as the conditions they value as the most significant for the overcoming of both their homelessness situation and other associated circumstances, such as alcoholism or drug dependence; and identifying the impact of these solidarity attitudes to their personal processes of recovery and general wellbeing. The next subsections show results structured according to these three core points.

### Negative Social Environments and Occultations: The Boundaries for the Transformation

When participants share their life stories, they perform an evaluation and a balance the factors that led them to the streets or to substance dependence. They share their life experiences while searching for answers that they themselves try to find along their narratives. In this pursuit of clues for understanding why things happened in that way and what helped them to get out of that situation, they found that there were some barriers that create a hindrance for the total transformation of their circumstances.

One of the boundaries that participants have recognized in their life stories is their decision of masking their reality, which hindered others to acknowledge their situation and the act of providing help. In the fear of entailing a burden, bothering their social network, being judged, or any other repercussions, some of the participants acknowledged that they preferred not to inform their relatives of their situation: “I have not told [family] what is my current situation. It would entail a problem rather than any other thing” (Mar). “[Daughters] have never known that I am this way” (Manuel).

The factor of “being alone,” without a network of family and/or friends for support, has also been evaluated as a boundary for overcoming homelessness and the illnesses resulting from substance abuse. For instance, Sergio’s problem is the lack of a family to sharing his difficulties with and a consequent lack of opportunities to be supported, and he believes that this circumstance has hindered and delayed his recovery process:

“It is difficult itself. I have few possibilities to get out of considering my situation, my family environment (I don’t have it), without family support, I don’t have it. My parents passed away. I don’t have siblings. I am a single son. I don’t have family support and it is very difficult…” (Sergio).

The last barrier identified has to do with the presence of a social environment, but it is a negative one in this case. The pressure felt by a toxic social group, which pushes to start or to continue consuming alcohol or drugs and other substances, is associated by participants with more difficulties to get out of the damaging spiral. Some participants recognize that they know the ethics, but, in practice, they feel unable or reluctant to say “no” when it comes to their acquaintances, and sometimes this is because the message for consuming is attractive: “You think they are your friends… Although you know that it is wrong [to consume], as they do it, [you think] ‘why I don’t too? It’s not that bad,’ and then you fall into the well” (Margarita). “[…] The friends told me ‘do you want to taste this [drug]? You will like it!’ […] Once, in the streets, they invited me” (Virginia).

### Significant Conditions for Overcoming Homelessness: Solidarity Attitudes and Networks

When participants communicated their experience of the process of overcoming their homelessness situation and the circumstances associated with it, there is a condition that always appeared in the life stories: the solidarity networks they found along the way. Although there are people who expressed that the solidarity performed by their family or friends had been the pillar of their recovery, most of them underlined that the improvement of their situation could not have been possible without the strong solidarity network of professionals and volunteers they had found along their path.

In terms of the support provided by the net of professionals, participants expressed how social workers, but also psychologists and doctors, had been a great help in their particular processes. Javier, who thought there was no solution for his alcohol addiction, happily said that he found the help he needed in the doctor of the entity where he was conducting his recovery program who encouraged him to trust his own capability to fight against the addiction and recognized his achievements: “I was lucky to find ‘the doctor’ […] I wonder how [I would stop drinking], because I don’t have willpower. The doctor says that it is the merit of mine, but I say that it is his merit” (Javier). For Emilio, everybody can find support in all the professionals of the entity, but he underlined the importance of being predisposed to being helped: “All the professionals help you […] If they don’t help you it is because you don’t accept the help” (Emilio). Virginia expressed her gratitude toward all the professionals, especially her social assistant, talking about them as a “big family”: “Rafael [social worker]… wow! Have you seen him? He is very kind, he is my family. Is he my support! The rest of them [the professionals of the entity] too […] It is the big family! (Virginia). In the case of Noelia, the solidarity and support performed by the entity’s workers enabled the transformation of much of her reality. She felt they have saved her from alcoholism and living on the street, and that they have provided her with the opportunity to get a new stable situation, to feel included in society, and to have a positive attitude toward life:

“In the entity they helped me a lot and they saved me […] I couldn’t [stop drinking] before, and they did it […] I started to have a normal life […] Everyone needs to feel included at society, and they distanced me from the negativism” (Noelia).

The solidarity performed by the volunteers has also been strongly acknowledged. For many participants, volunteer people have given their time to cover many basic needs (providing food, clothes, or blankets) but also to listen to them and providing emotional support; this was what many of the interviewees estimated as the most important kind of help. This support materialized in the form of the interviewees gaining confidence on their own capabilities, and they were trusted and encouraged: “The clue is that [the volunteers] gave me confidence” (Javier). Here Virginia stresses the solidarity and the help on the behalf of the volunteers, and the wonderful job they do: The volunteers of the street, they have helped me a lot, they supported me a lot […] They helped us, they listened to us… wow, very, very nice! Thanks to them who do such beautiful work” (Virginia).

Some of the participants went a step forward and revendicated the idea of investing in more professionals instead of other kinds of resources. As they stated, food is available and ensured, basic needs are already covered (in entities), but what they consider most significant, the support networks, should receive more attention. Manuel wondered why there are not more professionals working in entities considering that they are crucial in the processes, attending and helping to solve complex circumstances, such as the substance abuse problem: “Why are not there more job posts like that? […] Those who tell you ‘you have a problem with alcohol,’ and they solve it. They accompany you in the process […] It helps a lot!” (Manuel).

Regarding the role of friends and family, subjects reflected on how important the solidarity of their relatives has been in the process of overcoming their difficult situation. Thus, and in a complementary way, while participants were being attended by one or more of these entities, they counted on the collaboration of their inner circle, which made efforts to provide the help necessary to reach recovery or a stable situation. In this sense, Sonia expresses that her friend employed her for work, so she could have a salary, despite her poor financial situation: “My friend, who has a small shop, has hired me […] although things are not going well for her” (Sonia). In the case of Ana, her most significant cornerstones have been her mother as well as her significant other who has been a great support in her fight against alcoholism for years: “They are my two pillars […] Without them, I would not be alive, I wouldn’t have a life.”

According to the life stories, solidarity networks helped many participants to make homelessness more bearable during that previous stage of their lives. Even in those extreme and challenging circumstances, they acknowledged that they sometimes felt positive feelings, happiness even, due to the solidarity they experienced. For Sonia, to be homeless was not a disturbing experience because of the strong supporting network she had behind her while on the streets: “It has not been a traumatic experience. It has been a stage of my life where I have found people who have helped me!” (Sonia). Manuel also remembers the kindness of homeless mates and their willingness to help: “People at the streets helps each other a lot! I have seen many solidary people at the street […] Homeless people is very warm; they inform you about everything!” (Manuel). It is under these difficult homeless circumstances when the solidarity network of friends was a source of great support for some interviewees. Virginia said that different homeless mates joined together to share common spaces and resources and to help each other as a unit: “We joined together. I had good friends; we were a good team!” (Virginia). For Sergio, it was in these extreme conditions that he has realized who his actual friends were–the ones who had been available to him when he had nothing to offer: “I had three close friends, which are the ones I appeal in extreme situations […] It is [in those extreme circumstances] when you realize who are your real friends” (Sergio).

### The Impact of Solidarity: Consolidation of Social Networks, More Solidarity, and the Emergence of Feelings

Participants showed that the solidarity actions they experienced had an influence on their general wellbeing. Resulting from that, participants stressed their need for recognizing and thanking the network of professionals that were such a big support and also their willingness to contribute and toward other users.

One of the direct impacts of the solidarity demonstrations experienced by the participants was the reinforcement of that supportive social network, which started in a tough stage of their lives, and they looked for its maintenance and continuity. Many interviewees underlined how, at a time after their treatments and/or completing their intervention programs, they look for the social workers, psychologists, or other professionals who helped them in the process, in order to keep in contact, thank them, and to worry about them. Margarita said that she usually goes to the entity where her former social assistant works in order to meet her: “I went to meet her and we were talking […] I go there and ask about her” (Margarita). The way Teresa thanks all the permanent support received by professionals is cooking for them and inviting them for a meal: “I cooked chicken for them, and [the social worker] came […] They have always been there. They supported me when I was sad” (Teresa). Virginia expresses her gratitude toward volunteers, meeting them once a week and maintaining a close relationship with them: “I come [where volunteers are] once a week, just to meet them, and we have such a nice relationship with them… thanks, thanks, thanks to them who do a beautiful job!” (Virginia).

Another consequence of the solidarity support is the willingness of participants to contribute to the entity with their own work and effort, i.e., the development of more solidarity attitudes. Depending on their availabilities, some of them maintain regular collaboration as volunteers, while others look for the occasions for fixing and repair elements of the building according to their expertise. This has been the case of Javier, who feels the need to cooperate in order to show his gratitude for the assistance he received: “They helped you, you must collaborate too” (Javier). Estefania, apart from being a user, regularly participates as a volunteer, for tidying and cleaning rooms; her wish to help is a product that emerged after receiving a great support:

“I come from 4 p.m. until 8 p.m. […] I do laundry, I tidy the rooms […] I was helping the cleanser for a period […] and they offered me to be volunteer […] and I go there from 4 to 8 p.m.” (Estefania).

After experiencing the solidarity attitudes in the social environment of the entities, a new and strong network of relationships has been established, and feelings of love, appreciation, or friendship emerged toward those people who provided great support for the participants. In the words of Sonia, the help was much more than she could have expected, and it developed a sentiment of brotherhood, where they considered the professionals as part of their family–as essential figures of their overcoming process. Margarita, for her part, expresses how much she loves her social assistant, attributing to her the recuperation of enthusiasm for life and her second opportunity:

“Really, I couldn’t imagine [the reception], what these [professionals] do, like if they were your own family, like if you were a part of them. The siblings who give you a goodnight kiss […] Without them, I would not be overcome it” (Sonia).

“I love [my social worker]so much. I will thank her to the rest of my life; she has understood me, she has listened to me a lot. And she has understood me quite well […] Life starts for me again” (Margarita).

Like Margarita and Sonia, more participants have experienced a change in their lives. The impact of this solidarity environment is reflected on their words when they recognize how their lives have come back on track and how hope and passion have emerged again in their lives. Virginia not only feels that she has recovered a stable life as well as positive sentiments of optimism and enthusiasm: “I have moved off the streets and with hope and illusion” (Virginia). In the case of Ana, this solidarity networks and the strong relationships have reversed her disappointment with life to a fervent desire for living:

“I am very excited for being here […] And it is very beautiful because, otherwise, I would not have the fight for rising up every day […] Five months ago, I wanted to die and, now, look! I want to live!” (Ana).

As mentioned before, participants feel that the people from the entities, regarding professionals and users, are like a family. Coexistence is sometimes recognized as being complex, but many of the subjects feel the people who share housing with them are like new relatives who encourage them to perpetuate solidarity attitudes. For Manuel, coexistence is fine, and he consider his roommates to be like his family: “[Coexistence] is quite good! We are like a family” (Manuel). Sonia said the same, and she values the good coexistence with her mates, with whom she shares and enjoy common daily moments and who also show solidarity attitudes toward her in daily life:

“[Coexistence] goes pretty well because [my roommates] are quite nice! […] We have dinner together, we watch TV together… doors are always open. I had been working the whole month […] and I left the clothes inside the washing machine, and by the time I arrived I had my clothes hung out! They are very neat too” (Sonia).

## Discussion and Conclusion

Psychology has greatly improved the study of different elements and risk factors that cause or are related to homelessness. It has also improved identification of consequences of this extreme situation. Conversely, many psychology (and other social sciences) contributions have stressed the importance of quality social networks on wellbeing and health in general (Umberson et al., 2010; Gerino et al., 2017; Holt-Lunstad et al., 2017), underlining the importance of social support for coping with the homelessness situation and the circumstances associated to it (Paul et al., 2018; Durbin et al., 2019; Miller and Bowen, 2019). Solidarity, as a specific kind of social support that implies sacrifice, confidence, or trust, among other kinds of prosocial behaviors, can have a specific role in the overcoming of homelessness and its related problems. Therefore, when looking for a step forward in achieving a positive social impact through the research into the homeless population in their process of overcoming, it is necessary to (a) to take account the advances in psychology regarding the quality of social relationships for a better life and (b) to consider the end-users’ own views and experiences, which stress the importance of solidarity attitudes and networks in their overcoming processes. Communicative Methodology, used in this study, has allowed progress in this respect to move toward an integral understanding of the situation and a joint construction of transformative alternatives. As has been shown along the results section, in the process of sharing their life stories, participants’ dialogue showed how they balanced and evaluated their experiences, how they came to be in such a vulnerable situation, and how they searched for reasons and keys to understanding their realities. It is in that sense how Communicative Methodology contributed to the potential social impact of research, as it facilitates reflection on one’s live and identifying barriers and facilitators, which can serve to guide their futures.

What participants of this study have underlined as the most significant condition that has helped them to overcome their vulnerable situation, either homelessness itself or homelessness associated with substance addiction, has been the solidarity networks they found in their environment. Some of the participants have valued the solidarity performed by their close family, and others have stressed the role of friends, even the solidarity attitudes among mates on the streets. They have all focused on the significant solidarity demonstrations provided by the community of volunteers and professionals. Many of them have also stressed the fact that what they needed was not the supply for covering physiological needs but those solidarity actions of support, which had an impact on them on an emotional level. This fact is consistent with the scientific literature, especially with the late contributions of Maslow (1970); Wahba and Bridwell (1976), and Henwood et al. (2015) regarding the need for covering higher-level needs related to the self. In this regard, homelessness, which is a matter of lacking the coverage of basic physiological needs and safety (food, sleep, shelter, etc.), will also entail difficulties related to belonging, esteem, and self-actualization. It seems clear that the mentioned solidarity networks that understood confidence attitudes, encouragement, and appreciation were key to the social support provided for guaranteeing good coverage of the people demands. The need to strongly consider the inclusion of this condition in all the intervention programs and actions addressed to people who are in the process of overcoming homelessness is therefore emphasized.

We have seen that solidarity actions have a positive impact on perceived wellbeing, a development of more solidarity attitudes as a permanent willingness to help others as well, but also the development and maintenance of ties of friendship or the emergence of feelings of affection, gratitude, and a positive attitude and enthusiasm toward life. These impacts would take a step beyond to the recent literature regarding how social support from family, friends, and professionals (Paul et al., 2018) has been related to higher levels of resilience (Durbin et al., 2019). Evidence seems consistent with the literature on the importance of the social environment on alcohol and drug abuse (Kennedy et al., 2017), which has also been revealed in the research, such as how a negative social environment directly pressures people into getting involved in substance abuse. This study has brought into the conversation further evidence for overcoming a homeless situation and the circumstances associated with it. The most clear conclusions seems to be that (a) solidarity networks have a powerful impact on the process of reaching the stable situation desired, and (b) a supportive and empowering solidarity network is crucial to overcoming those negative circumstances and consequences related to or derived from homelessness.

Despite the difficulties of interviewing people in a situation of homelessness, 20 people voluntarily agreed to be interviewed. However, it is also necessary to consider the limitations of this study. The study could be deepened further with more interviews on other factors that have led people, especially women since they are one of the most vulnerable groups, to find themselves in a situation of homelessness, such as having suffered gender-based violence. Likewise, information was gathered in a specific period of their lives. Further longitudinal studies focused on the transformative role of solidarity and positive communitarian environments would be of interest in order to check the evolution of the people’s circumstances. Action and intervention programs that provide an integrated approach, considering not only the potential of a quality social context but also putting emphasis on counting on a strong and continuous solidarity network, seem necessary to tackle the multidimensional problem of homelessness in pursuit of a potential social impact on these people’s lives. The development of psychological intervention programs, including the emotional perspective regarding solidarity and friend networks, would be useful in the path for improving homeless people’s lives from the perspective of psychology and other social sciences. Testing, with different samples (in terms of age, precedence, status, etc.), the efficacy of including these conditions on intervention programs would therefore advance the knowledge and social impact of psychological research.

## Data Availability Statement

The datasets generated for this study are available on request to the corresponding author.

## Ethics Statement

The studies involving human participants were reviewed and approved by Ethics Committee of the Community of Researchers on Excellence for All, University of Barcelona. The patients/participants provided their written informed consent to participate in this study.

## Author Contributions

VM-D was responsible for the field work of the project. AM-P, VM-D, and ID developed the manuscript in relation to social work and the attention to homeless individuals and performed the data analysis. SL-J collaborated in the data analysis and in the final writing of the manuscript. AM-P revised and approved the submitted version.

## Conflict of Interest

The authors declare that the research was conducted in the absence of any commercial or financial relationships that could be construed as a potential conflict of interest.

## Footnotes

1 The Ethics Board was composed by: Dr. Marta Soler (president), who has expertise within the evaluation of projects from the European Framework Program of Research of the European Union and of European projects in the area of ethics; Dr. Teresa Sordé, who has expertise within the evaluation of projects from the European Framework Program of Research and is a researcher of Roma studies; Dr. Patricia Melgar, a founding member of the Catalan Platform against gender violence and researcher within the area of gender and gender violence; Dr. Sandra Racionero, a former secretary and member of the Ethics Board at Loyola University Andalusia (2016–2018) and review panel member for COST action proposals in the area of health; Dr. Cristina Pulido, an expert in data protection policies and child protection in research and communication and researcher of communication studies; Dr. Oriol Rios, a founding member of the “Men in Dialogue” association, a researcher within the area of masculinities, as well as an editor of “Masculinities and Social Change,” a journal indexed in WoS and Scopus; and Dr. Esther Oliver, who has expertise within the evaluation of projects from the European Framework Program of Research and is a researcher within the area of gender violence.
